# LncRNA H19 governs mitophagy and restores mitochondrial respiration in the heart through Pink1/Parkin signaling during obesity

**DOI:** 10.1038/s41419-021-03821-6

**Published:** 2021-05-28

**Authors:** Shao-Hua Wang, Xiao-Lin Zhu, Fei Wang, Si-Xu Chen, Zhi-Teng Chen, Qiong Qiu, Wen-Hao Liu, Mao-Xiong Wu, Bing-Qing Deng, Yong Xie, Jing-Ting Mai, Ying Yang, Jing-Feng Wang, Hai-Feng Zhang, Yang-Xin Chen

**Affiliations:** 1 Department of Cardiology, Sun Yat-sen Memorial Hospital, Sun Yat-sen University, 510120 Guangzhou, Guangdong China; 2 Laboratory of Cardiac Electrophysiology and Arrhythmia in Guangdong Province, 510120 Guangzhou, Guangdong China; 3 Guangzhou Key Laboratory of Molecular Mechanism and Translation in Major Cardiovascular Disease, Sun Yat-sen Memorial Hospital, Sun Yat-sen University, 510120 Guangzhou, Guangdong China; 4 Department of Anesthesiology, Sun Yat-sen Memorial Hospital, Sun Yat-sen University, 510120 Guangzhou, Guangdong China

**Keywords:** Mitophagy, Long non-coding RNAs, Obesity

## Abstract

Maintaining proper mitochondrial respiratory function is crucial for alleviating cardiac metabolic disorders during obesity, and mitophagy is critically involved in this process. Long non-coding RNA H19 (H19) is crucial for metabolic regulation, but its roles in cardiac disorders, mitochondrial respiratory function, and mitophagy during obesity are largely unknown. In this study, palmitic acid (PA)-treated H9c2 cell and Lep^−/−^ mice were used to investigate cardiac metabolic disorders in vitro and in vivo, respectively. The effects of H19 on metabolic disorders, mitochondrial respiratory function, and mitophagy were investigated. Moreover, the regulatory mechanisms of PA, H19, mitophagy, and respiratory function were examined. The models tested displayed a reduction in H19 expression, respiratory function and mitochondrial number and volume, while the expression of mitophagy- and Pink1/Parkin signaling-related proteins was upregulated, as indicated using quantitative real-time PCR, Seahorse mitochondrial stress test analyzer, transmission electron microscopy, fluorescence indicators and western blotting. Forced expression of H19 helped to the recoveries of respiratory capacity and mitochondrial number while inhibited the levels of mitophagy- and Pink1/Parkin signaling-related proteins. Pink1 knockdown also attenuated PA-induced mitophagy and increased respiratory capacity. Mechanistically, RNA pull-down, mass spectrometry, and RNA-binding protein immunoprecipitation assays showed that H19 could hinder the binding of eukaryotic translation initiation factor 4A, isoform 2 (eIF4A2) with Pink1 mRNA, thus inhibiting the translation of Pink1 and attenuation of mitophagy. PA significantly increased the methylation levels of the H19 promoter region by upregulation Dnmt3b methylase levels, thereby inhibiting H19 transcription. Collectively, these findings suggest that DNA methylation-mediated the downregulation of H19 expression plays a crucial role in cardiomyocyte or H9c2 cells metabolic disorders and induces cardiac respiratory dysfunction by promoting mitophagy. H19 inhibits excessive mitophagy by limiting Pink1 mRNA translation, thus alleviating this cardiac defect that occurs during obesity.

## Introduction

Obesity is a worldwide health problem imposing risk for many serious diseases, particularly cardiovascular disease, which is the leading cause of death all around the world^1,2^. The increase in body weight causes a series of typical cardiomyopathy events, which correlate with high risk of sudden cardiac death^3^. Besides, chronic obesity directly impairs cardiac structure and function^4,5^. Obesity-induced cardiac impairments are considered to occur due to the alterations in myocardial lipid metabolism^6,7^. Other factors, such as mitochondrial dysfunction, autophagy, and inflammation, have also been reported to be critically involved^8^. However, the precise cues of obesity-induced cardiac function impairment remain elusive.

Several studies have linked the abnormal autophagy to cardiovascular damage in obesity. We and others have shown that saturated fatty acids are important for regulating non-selective cardiac autophagy and thus determining cell fate^9,10^. Recently, mitophagy, a selective form of autophagy that eliminates the impaired mitochondria *via* an autophagic mechanism^11^, has been reported to be implicated in cardiovascular disorders^12,13^. Mitophagy is triggered by the decrease of mitochondrial membrane potential and is the main mechanism that regulates mitochondrial quality and quantity^14^. Although mitophagy is traditionally thought to serve as a protective mechanism, it has also been accepted that while moderate mitophagy maintains mitochondrial health and preserves energy production, excessive mitophagy in the absence of mitochondrial biogenesis is detrimental to cells^15–18^. Therefore, the detailed roles of mitophagy are still under sharply debate. In addition, there is limited information available on the mechanisms that trigger mitophagy in the heart during obesity, making it difficult to explore effective therapeutic interventions to some extent.

Long non-coding RNAs (lncRNAs) are a type of RNA with transcripts exceeding 200 nucleotides that are not translated into proteins. Although the importance of lncRNAs in cardiac physiological and pathological processes are recognized, their roles in mitophagy have not yet been explored in detail. Among the lncRNAs potentially involved in obesity-induced cardiac function, lncRNA H19 (H19) warrants investigation. Some studies have reported that H19 alleviates cardiac defects by inhibiting mitochondrial apoptosis^19^ or suppressing the levels of cardiac inflammatory cytokines in high-fat diet-fed mice^20^. In addition, H19 has been shown to participate in cardiac function management in an autophagy-related manner^21^. However, the roles of H19 in mitochondrial mitophagy and function, which is critically involved in obesity-induced cardiac damage, are unknown.

Therefore, in the current study, we performed in vivo and in vitro analyses to investigate the roles of H19, mitophagy, and mitochondrial respiratory function in the heart during obesity. Moreover, we clarified the detailed mechanisms involved in the H19-mediated modification of cardiac function and mitochondrial respiration, as well as the regulation of H19 expressions.

## Results

### Poor expression of lncRNA H19 was responsible for the reduced mitochondrial respiration and cardiac dysfunction in obesity

**Fig. 1:**
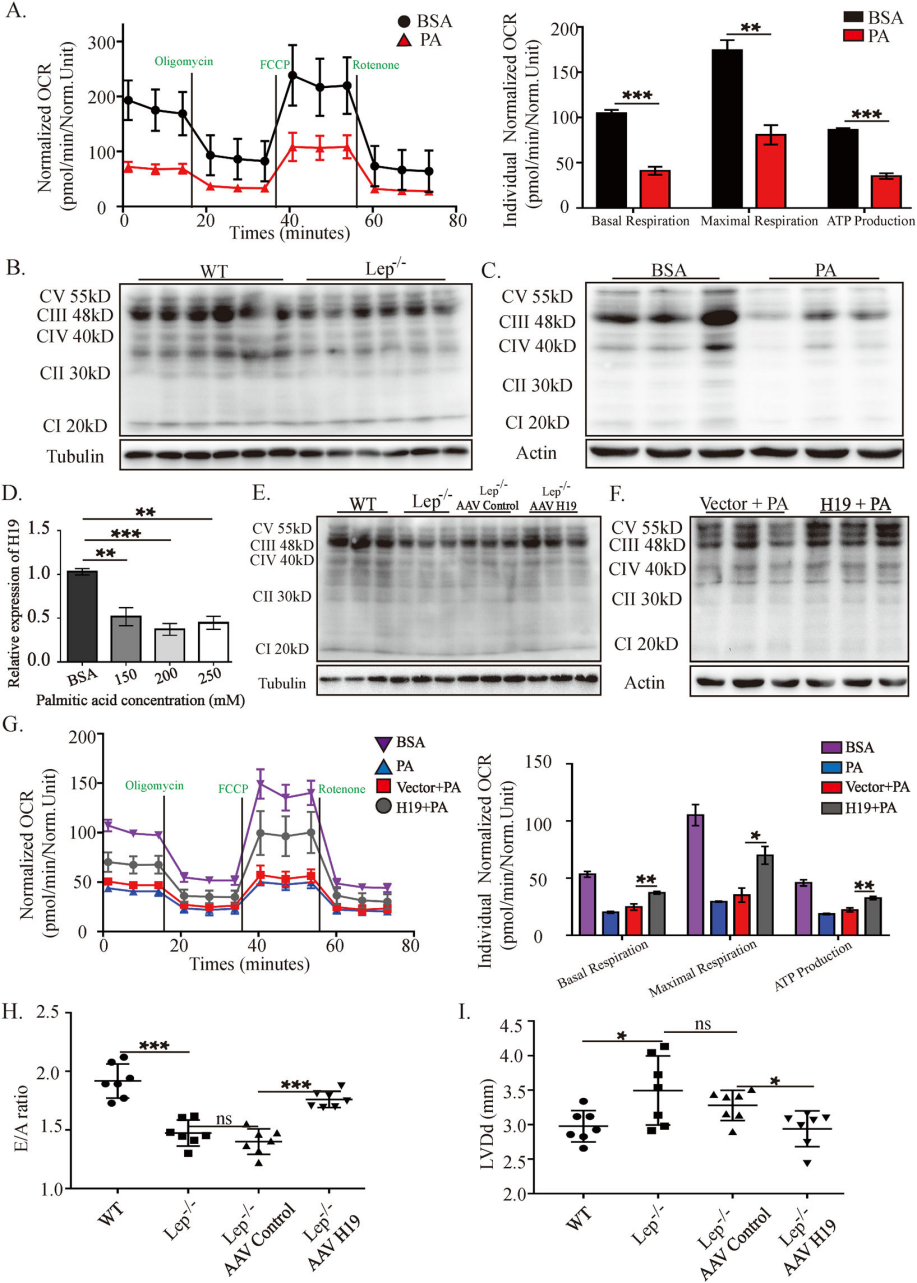
Poor expression of lncRNA H19 was responsible for the reduced mitochondrial respiration and cardiac dysfunction in obesity. **A** Seahorse profile for OCRs of the two groups treated with 1.5 μM oligomycin, 0.5 μM FCCP, and 1.5 μM antimycin/rotenone. **B**, **C** Western blots of mitochondrial electron transport chain complexes (complexes I–V) in vivo (*n* = 6) and in vitro (*n* = 3). Results of optical density analyses were presented in Sup. Fig. 1A, B. **D** RT-qPCR for detection of H19 levels upon treatment with various concentrations of PA in vitro. **E**, **F** Western blot analysis of mitochondrial electron transport chain complexes (complexes I–V) following H19 overexpression in vivo (*n* = 6) and in vitro (*n* = 3). Results of optical density analyses were presented in Sup. Fig. 1C, D. **G** Seahorse analyses for OCR in H9c2 cells treated by BSA, PA, control lentivirus (vector + PA), and H19 lentivirus (H19 + PA). **H** and **I** Echocardiography assessments of E/A ratio and LVDd values in the different treatment groups of Lep^−/−^ mice, *n* = 7 per group; Data in **A**–**I** are expressed as mean ± SEM. *P < 0.05; **P < 0.01; ***P < 0.001. OCR, oxygen consumption rate; BSA, bovine serum albumin; PA, palmitic acid; CI-CV, mitochondrial respiratory chain complex I–V; Lep^−/−^, B6-OB mice with leptin defects; WT, littermate control of Lep^−/−^; AAV, adeno-associated virus; RT-qPCR, quantitative reverse transcription polymerase chain reaction; LVDd, left ventricular end-diastolic dimension; ATP, adenosine-triphosphate.

Mitochondrial respiratory function, as represented by basal/maximal respiration capacity and adenosine-triphosphate (ATP) production, analyzed using the Seahorse XF mitochondrial stress test analyzer, was significantly reduced in H9c2 cells upon palmitic acid (PA) administration (Fig. 1A). All mitochondrial respiratory complexes were determined under these conditions and results are displayed in Fig. 1B, C. As shown in Fig. 1B, in vivo study revealed that compared to the control mice, the expression of complexes II, III, and IV was significantly reduced in Lep^−/−^ mice. The contents of complex III were profoundly higher than those of other complexes (Fig. 1B and Sup. Fig. 1A). Results from H9c2 cells strengthened these findings, showing a consistent reduction in the levels of all the complexes in PA-treated cells, with complex III still being the most abundant (Fig. 1C and Sup. Fig. 1B).

As for H19 expressions, the results showed that in the Lep^−/−^ genetic model mice, the cardiac expression of H19 was reduced to 50% of that in the control mice (Sup. Fig. 1C). H9c2 cells were treated with different doses of PA, and the results showed a similar reduction in H19 expression (Fig. 1D). We next explored the relationship between barren H19 expression and mitochondrial respiration suppression. Forced expression of H19 using adeno-associated virus (AAV) in Lep^−/−^ mice under a cardiomyocyte-specific promoter, cardiac troponin T (cTnT), resulted in a marked increase in complex III levels, in addition to moderate elevations in the levels of complexes IV and V (Fig. 1E and Sup. Fig. 1D). Similar results were observed in PA-treated H9c2 cells (Fig. 1F and Sup. Fig. 1E). Results from H9c2 cells also showed that H19 could rescue the respiratory capacity, as manifested by the consistent changes of basal respiration, maximal respiration, and ATP production (Fig. 1G).

We next investigated whether restoring H19 expression could alleviate cardiac dysfunction in the setting of obesity. The altered *E*/*A* ratio and left ventricular end-diastolic dimension (LVDd), which respectively represent left ventricular diastolic function and cardiac remodeling, were completely restored upon cardiomyocyte-specific expression of H19 (Fig. 1H, I). The full data of the echocardiography assessment are provided in Sup. Fig. 1F.

These results indicated that suppressed the expression of H19 in cardiomyocytes or H9c2 cells are critical for cardiac dysfunction and impaired mitochondrial respiration in obesity.

### Forced expression of H19 rescues cardiac mitochondrial mass reduction

**Fig. 2:**
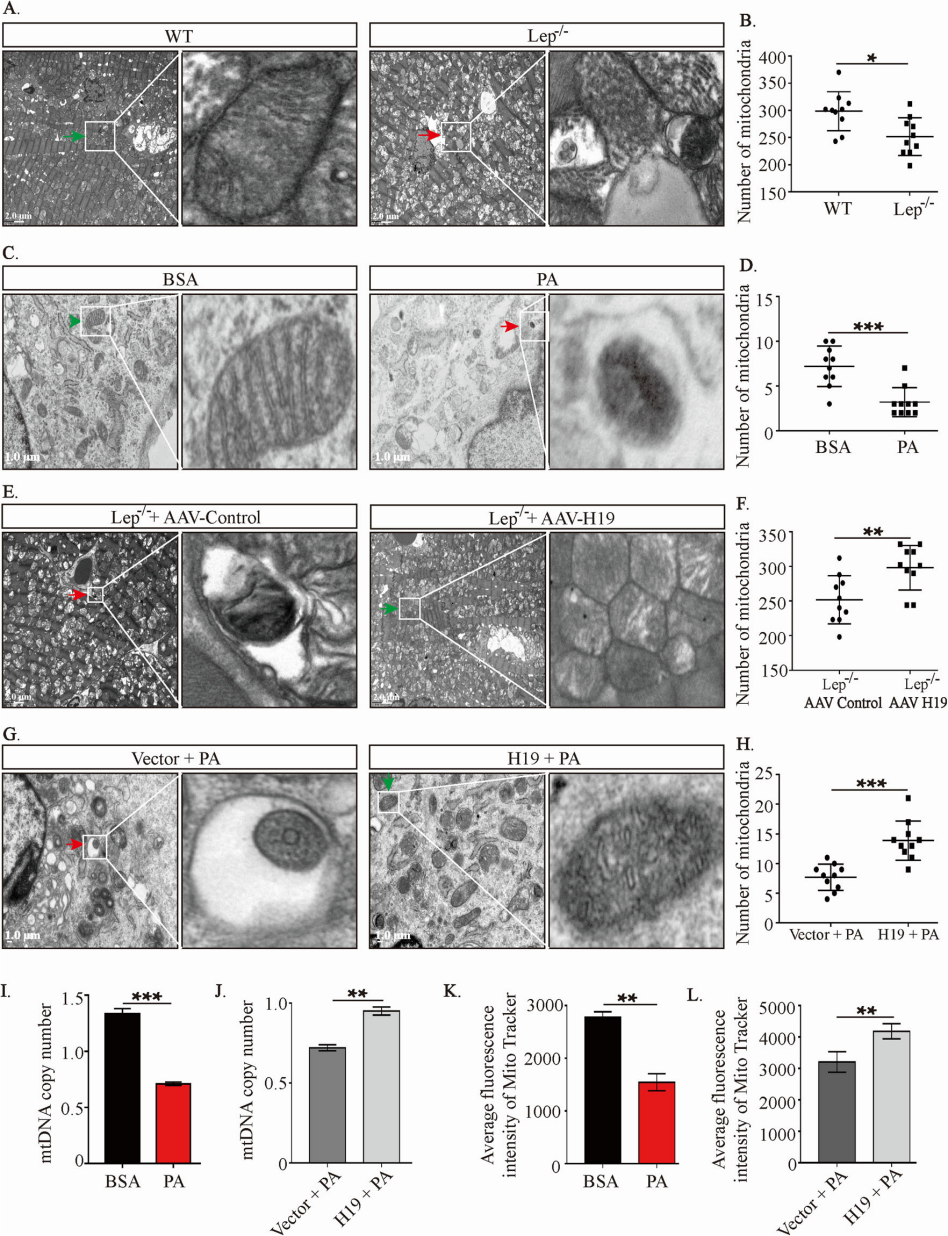
Forced expression of H19 rescues cardiac mitochondrial mass reduction. **A**–**H** Morphologies and number of the mitochondria in H9c2 cell and left ventricular of mice detected by TEM under different treatments. Red arrows indicated autophagosomes or autolysosomes, including degraded mitochondria, while green arrows indicate normal mitochondria. The zoomed-in images represented high-magnification views of the outlined areas. Ten images were obtained for each group. Scale bar: 2 μm (in vivo) and 1 μm (in vitro). **I**–**L** Effects of PA and H19 on mitochondrial number in H9c2 cells assessed by mtDNA copy number (detected by PCR) and MitoTracker™ Green dye staining (detected by flow cytometry). Data in **A**–**F** are expressed as mean ± SEM. *P < 0.05; **P < 0.01; ***P < 0.001. TEM, transmission electron microscope; BSA, bovine serum albumin; PA, palmitic acid; Lep^−/−^, B6-OB mice with leptin defects; WT, littermate control of Lep^−/−^; H19, H19 overexpression; Vector, control lentivirus of H19; AAV, adeno-associated virus; mtDNA, mitochondrial DNA.

Mitochondria are the main sites for oxidative respiration, and therefore, we assessed the quantity and morphology of mitochondria. Transmission electron microscopy **(**TEM) was used to directly observe mitochondrial number and morphology. As shown in Fig. 2A, B, compared to mitochondrial numbers in the ventricular tissues of the control mice, those in the ventricles of Lep^−/−^ mice were reduced to only about 60%. In addition, mitochondria in the heart of these mice showed obvious shrinkage (Fig. 2A, B). H9c2 cells treated with PA exhibited similar changes (Fig. 2C, D). Notably, cardiomyocyte-specific restoration of H19 expression increased in about 35 and 60% of mitochondria in Lep^−/−^ mice and H9c2 cells treated with PA, respectively (Fig. 2E–H). Quantitative reverse transcription polymerase chain reaction (RT-qPCR) results showed that mitochondrial DNA (mtDNA) levels decreased to approximately 50% in PA-treated H9c2 cells, which was significantly reversed upon H19 expression (Fig. 2I, J). Staining using MitoTracker™ Green, a mitochondrial probe detected using flow cytometry, showed similar results, with ~40% reduction in green fluorescence intensity in PA-treated cells, which could be largely restored upon H19 expression (Fig. 2K, L).

These results demonstrated that suppressed H19 expression is a key event in obesity-induced mitochondrial mass reduction.

### H19 attenuates PA-induced mitophagy enhancement in H9c2 cells

**Fig. 3:**
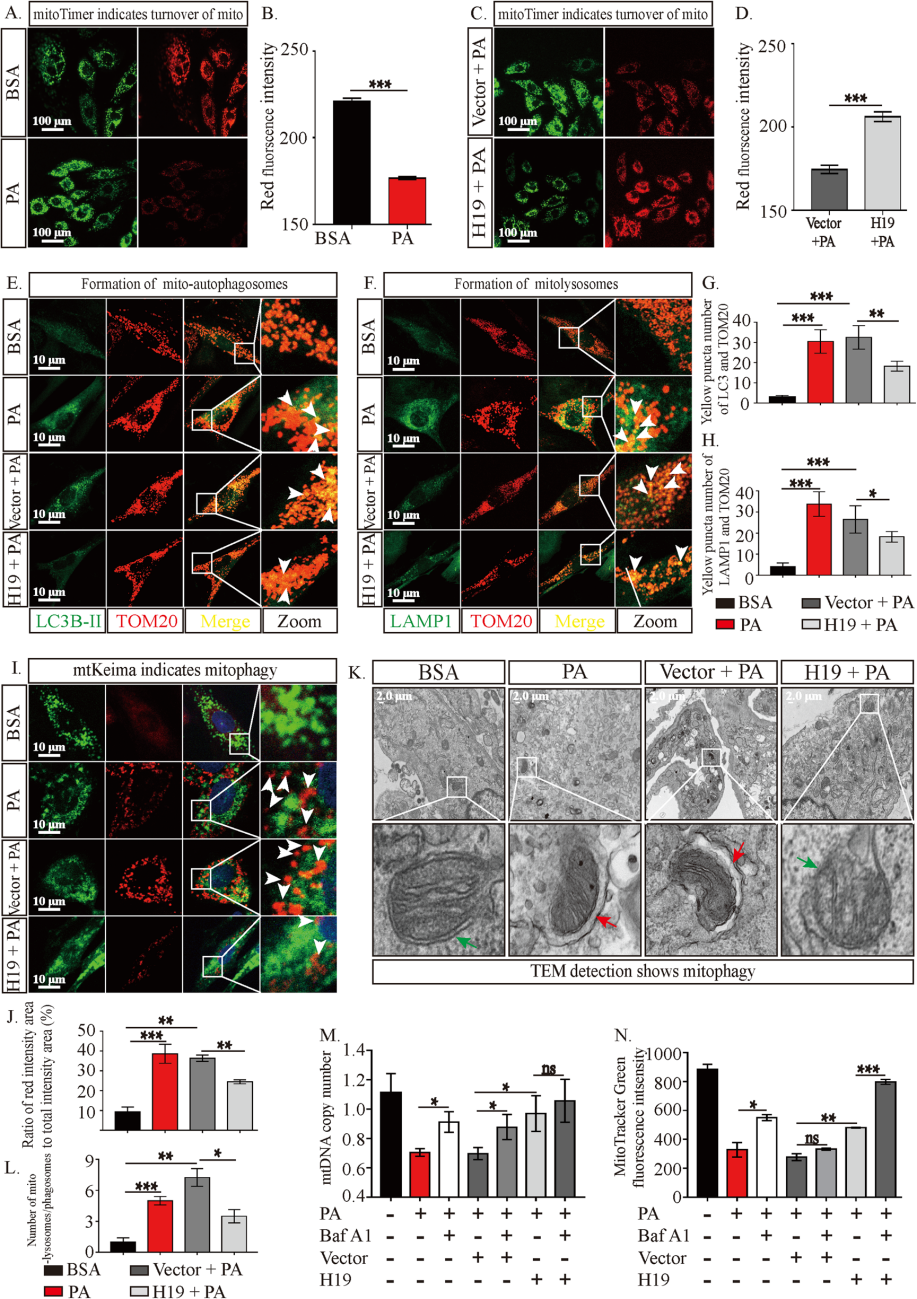
H19 attenuates PA-induced mitophagy enhancement in H9c2 cells. **A**–**D** Effects of PA and H19 on mitochondrial turnover assessed by mitoTimer. The initial state of this protein is green, which could shift to red over time if mitochondria were not eliminated. Scale bar: 100 μm. **E** and **F** Effects of PA and H19 on the co-localization of mitochondrial marker (TOM20) and autophagosomes (**E**, LC3B-II)/autolysosomes (**F**, LAMP1) assessed by immunofluorescence staining. Yellow dots indicated co-localization of LC3B-II or LAMP1 and TOM20. PA treatment increased the number of yellow dots, and this trend was abolished upon treatment with H19. Scale bar: 10 μm. **G**, **H** Statistical analysis of yellow puncta numbers in (**E**) and (**F**), *n* = 4. **I** Green dots represent the initial state of mtKeima proteins or mitochondria, and red dots (some could be yellow) indicate mtKeima or mitochondria in lysosomes. A relatively increased ratio of red intensity area to total intensity area (red and green) suggested enhanced mitophagy. Scale bar: 10 μm. **J** Ratio of red intensity area to total intensity area (red and green) of mtKeima in **I**, *n* = 4. **K** The TEM images show normal mitochondria or mitophagy in H9c2 cells with different treatments. Green arrows represent normal mitochondria, and red arrows indicate mitochondria in autophagosomes or autolysosomes. The zoomed-in images show high-magnification views of the outlined areas Scale bar: 2 μm. **L** Statistical analysis of the number of mitochondria in autophagosomes or autolysosomes among the four groups, *n* = 4. **M** and **N** Effects of PA, H19 and bafA1 (autophagy inhibitor) on mitochondrial number assessed by mtDNA copy number (detected by PCR) and MitoTracker™ Green dye staining (detected by flow cytometry). Data in **B**, **G**, **H**, **J**, **L**, **M**, and **N** are expressed as mean ± SEM. All the statistical analyses were performed base on four randomly selected scopes under observed by confocal microscope (**A**–**J**) or TEM (**K**, **L**). *P < 0.05; **P < 0.01; ***P < 0.001. BSA, bovine serum albumin; PA, palmitic acid; H19, H19 overexpression; Vector, control lentivirus of H19; LC3B-II, light chain 3, mark of autophagosomes; TOM20, translocase of the outer membrane 20, mark of mitochondria; LAMP1, lysosome-associated membrane protein 1, mark of lysosomes; mtDNA, mitochondrial DNA; BafA1, bafilomycin A1.

Reduced mitochondrial number could be caused by either enhanced degradation or impaired biogenesis. Therefore, we aimed to determine alterations in mitophagy (the main pathway of mitochondrial degradation) and biogenesis. Peroxisome proliferator-activated receptorγcoactivator-1 and mitochondrial transcription factor A are the main proteins controlling mitochondrial biogenesis, and their expression was significantly increased in H9c2 cells upon PA treatment (Sup. Fig. 2A). In contrast, PA treatment markedly reduced the red fluorescence and slightly enhanced the green fluorescence in H9c2 cells carrying mitoTimer (Fig. 3A, B), which demonstrated a shortened existence duration of the mitochondria, implying enhanced mitochondrial degradation. H19 overexpression reversed this trend, indicating attenuation of mitophagy (Fig. 3C, D). Immunofluorescence staining showed enhanced LC3 (Fig. 3E) and LAMP1 (Fig. 3F) expression in PA-treated cells, which were both co-localized with the mitochondrial marker TOM20 (yellow dots, Fig. 3E–H). The number of yellow puncta was reduced upon H19 overexpression, which implied an attenuation of mitophagy level (Fig. 3E–H). The incidence of autophagy in mitochondria was further supported by the mitophagy tracker mtKeima. As shown in Fig. 3I, J, PA administration significantly increased the red intensity area in the cells, indicating enhanced autophagic flux in the mitochondria. This enhanced autophagic flux was significantly attenuated in H9c2 cells treated with H19 (Fig. 3I, J). TEM results showed an increased number of autophagosomes or autolysosomes carrying mitochondria in PA-treated cells. Additionally, morphological disorders of mitochondria were observed under these conditions. Similar to the former results, H19 overexpression in H9c2 cells reduced the upregulated levels of mitophagy, and ameliorated the disordered mitochondrial morphology (Fig. 3K, L).

To demonstrate the role of mitophagy in regulating the mitochondrial number, we used bafilomycin A1 (BafA1), a lysosomal inhibitor, and measured mitochondrial number by mtDNA copy number detection and MitoTracker™ Green staining. As shown in Fig. 3K, L, BafA1 rescued the decreasing trend of mitochondrial numbers (Fig. 3M, N and Sup. Fig. 1G).

These results showed that mitophagy was significantly enhanced during PA stimulus. The reduction in the number of mitochondria during these circumstances could be caused by increased mitophagy, instead of alterations in mitochondrial biogenesis.

### Pink1, which is downregulated by H19, is essential for mitophagy increase and mitochondrial respiration reduction

**Fig. 4:**
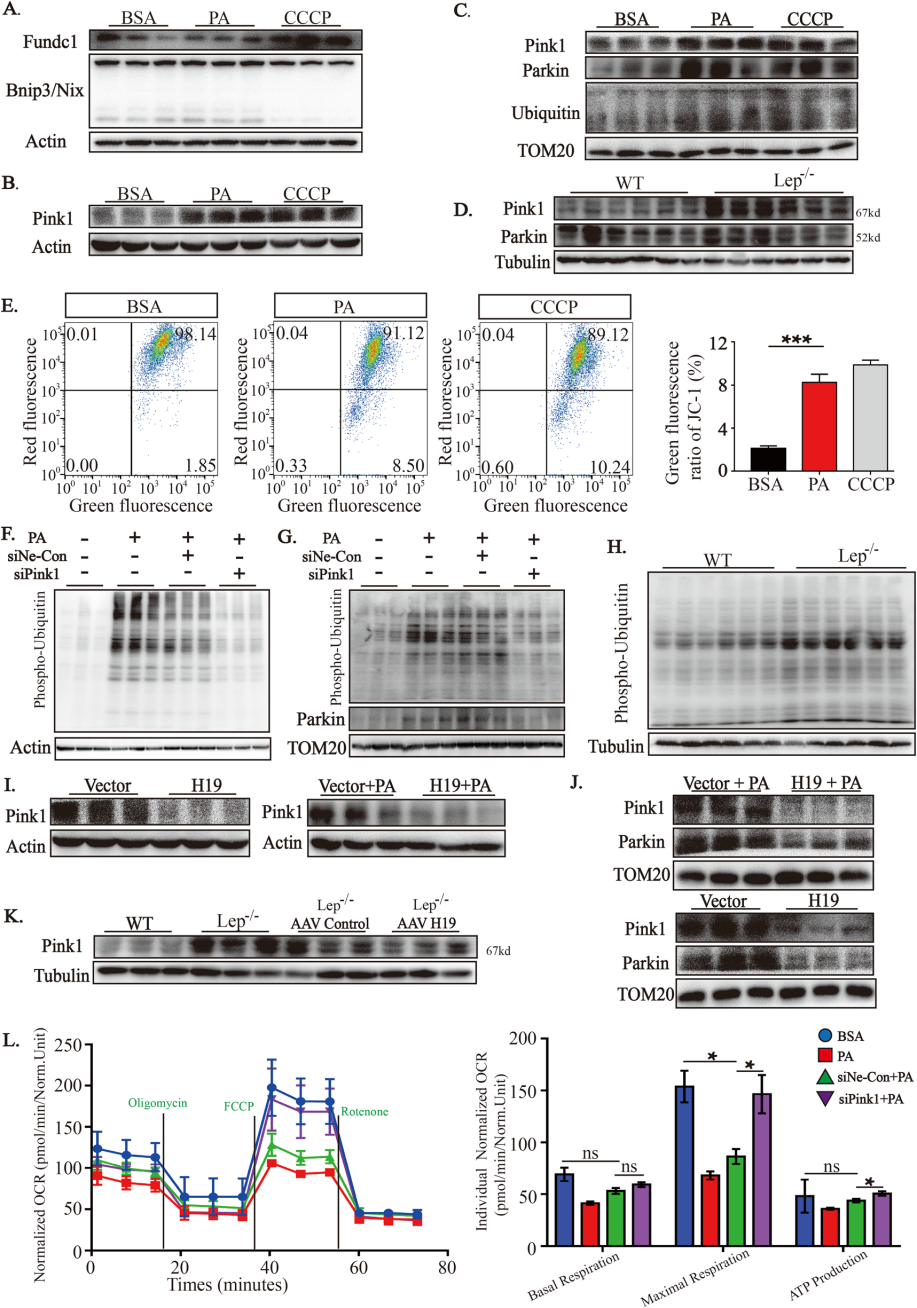
Pink1, which is downregulated by H19, is essential for mitophagy increase and mitochondrial respiration reduction. **A**, **B** Effects of PA and mitophagy inducers (CCCP) on Fundc1 (**A**), Binp3/Nix (**A**) and Pink1 expressions (**B**) in whole cell lysates (*n* = 3). Results of optical density analyses were presented in Sup. Fig. 2B, C. **C** Effects of PA on contents of Pink1/Parkin and ubiquitination levels in mitochondrial proteins of H9c2 cells (*n* = 3). Results of optical density analyses were presented in Sup. Fig. 2D. **D** Pink1/Parkin contents in WT or Lep^−/−^ mice (*n* = 6). Results of optical density analyses were presented in Sup. Fig. 2E. **E** Representative images of JC-1 probe detected by flow cytometry in H9c2 cells with different treatments. Green signal indicates mitochondrial membrane potential depolarization. Statistical analyses were performed base on three biological replicates. **F**, **G** Effects of PA and siRNA against Pink1 on the levels of phospho-ubiquitin in the whole cell and isolated mitochondrial proteins in H9c2 cells (*n* = 3). Results of optical density analyses were presented in Sup. Fig. 2G, H. **H** Levels of phospho-ubiquitin in whole cell lysates from left ventricular of WT or Lep^−/−^ mice (*n* = 6). Results of optical density analyses were presented in Sup. Fig. 2I. **I**–**K** Contents of Pink1 or Parkin proteins in whole cell lysates or isolated mitochondria from H9c2 cells with different treatment (**I**), (**J**) and different mice (**K**) (*n* = 3). Results of optical density analyses were presented in Sup. Fig. 2K–M. **L** Seahorse exhibited the alteratons of respiratory capacity following treatments with PA and siRNA against Pink1. Data in **E** and **L** are expressed as mean ± SEM. *P < 0.05; **P < 0.01; ***P < 0.001. BSA, bovine serum albumin; PA, palmitic acid; CCCP, mitophagy inducer; TOM20, translocase of the outer membrane 20, mark of mitochondria; Lep^−/−^, B6-OB mice with leptin defects; WT, littermate control of Lep^−/−^; si-Pink1, small interference RNA against Pink1; si-NE-Con, negative control of si-Pink1; H19, H19 overexpression; Vector, control lentivirus of H19; OCR, oxygen consumption rate.

It is widely accepted that mitophagy can be triggered by three canonical pathways, namely Pink1/Parkin, Binp3/Nix, and Fundc1^14,22,23^ and we explored the change of each. The in vitro experiments showed that upon PA treatment, there was no change in the expression of Bnip3/Nix (Fig. 4A and Sup. Fig. 2B). Fundc1 expression displayed a tendency to increase but no statistically significant could be found (Fig. 4A and Sup. Fig. 2B). However, there was profound upregulation in the expression of Pink1 in the lysate from whole cells or mitochondria (Fig. 4B, C and Sup. Fig. 2C, D). A significant increase was also noted in the levels of Parkin protein, an E3 ligase that can be recruited to mitochondria by Pink1, in the mitochondria isolated from PA-treated H9c2 cells (Fig. 4C and Sup. Fig. 2D). In according to this, ubiquitination levels of total mitochondrial proteins were also significantly increased (Fig. 4C and Sup. Fig. 2D). In vivo experiments using Lep^−/−^ mice supported the changes of Pink1 and Parkin (Fig. 4D and Sup. Fig. 2E).

Pink1 is specifically activated by mitochondrial membrane potential depolarization, which further enables parkin phosphorylation and triggers mitochondrial ubiquitination degradation. Therefore, to obtain more evidence supporting Pink1 activation, we assessed the membrane potential and levels of phospho-ubiquitin in proteins. As indicated in Fig. 4E, membrane potential depolarization was decreased in PA-treated cells. In accordance with these results, there was a significant increase in phospho-ubiquitin levels in both total proteins or mitochondrial proteins in PA-treated H9c2 cells and Lep^–/–^ mice (Fig. 4F–H and Sup. Fig. 2G–I). Moreover, knockdown of Pink1 almost completely abolished these effects (Fig. 4F, G and Sup. Fig. 2G, H). Collectively, these results showed that Pink1/Parkin signaling, but not Fundc1 or Bnip3/Nix, was crucial for enhanced mitophagy in the heart or H9c2 cells in metabolic disorders during obesity.

Next, we investigated whether Pink1/Parkin signaling was regulated by H19. In H9c2 cells, H19 dependently decreased Pink1 expression with or without PA treatment, in whole cell lysate or mitochondrial proteins (Fig. 4I, J and Sup. Fig. 2K, L). The contents of mitochondrial Parkin were also significantly decreased upon H19 expression (Fig. 4J). In the in vivo experiment, cardiac-specific overexpression of H19 significantly diminished Pink1 expression in left ventricular of Lep^−/−^ mice (Fig. 4K and Sup. Fig. 2M).

To further test the roles of Pink1 supprssing in mitochondrial respiratory function, we employed siRNA against Pink1^24,25^. As shown in Fig. 4L and Sup. Fig. 2J, Pink1 knockdown significantly restored PA-mediated inhibition of mitochondrial respiration and increased ATP production.

Altogether, these results showed that H19 could inhibit the Pink1/Parkin signaling pathway, which is the main pathway involved in inducing excessive mitophagy and reducing mitochondrial respiratory function.

### H19 interacts with eIF4A2 protein and inhibits Pink1 mRNA translation

The above results clearly demonstrated the opposite roles of Pink1 and H19 in obesity and PA-induced excessive cardiac mitophagy and identified that H19 negatively regulates Pink1 expression. However, the mechanism underlying the H19-mediated regulation of Pink1 is unknown.

**Fig. 5:**
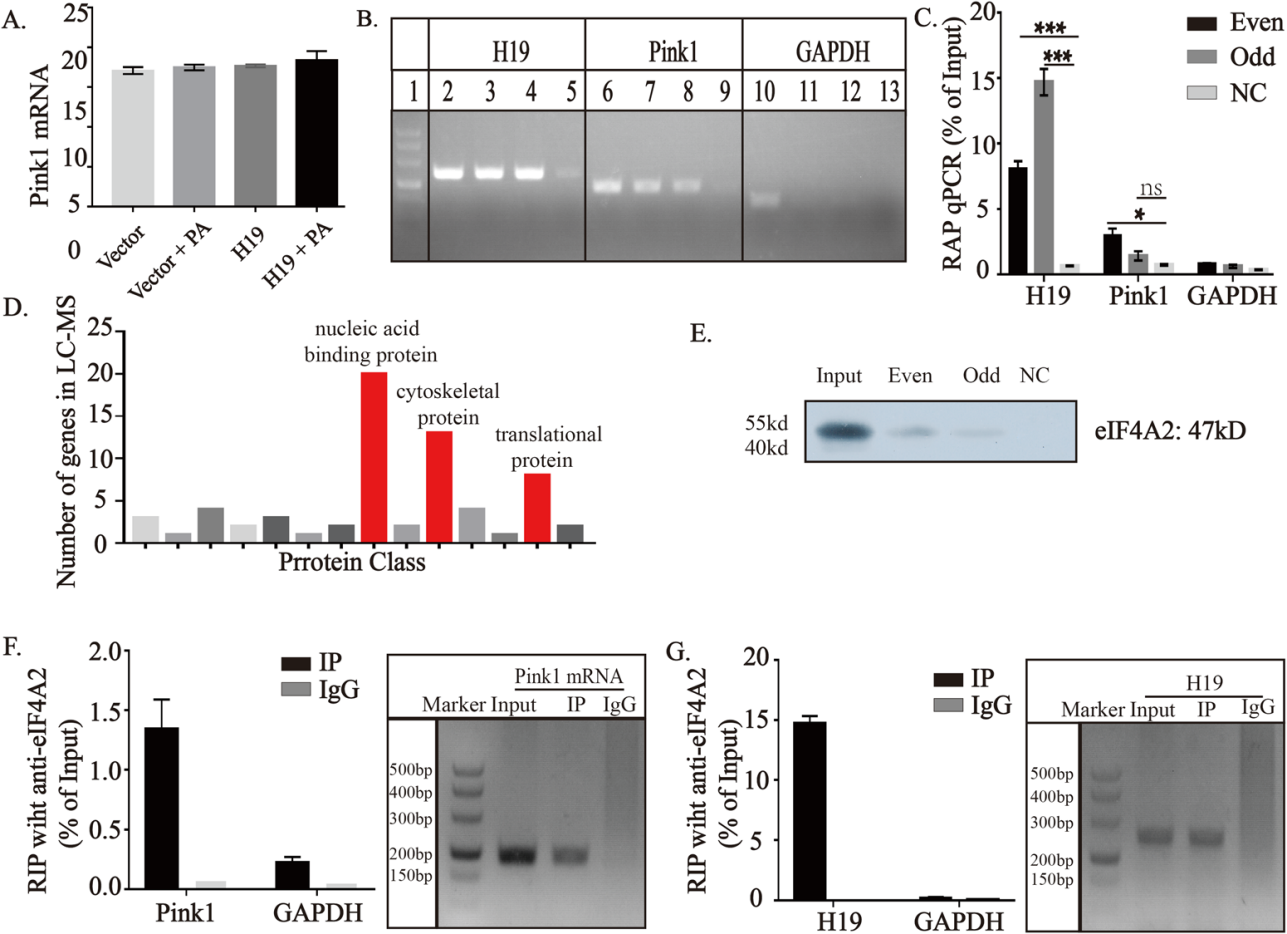
H19 interacts with eIF4A2 protein and inhibits Pink1 mRNA translation. **A** Effects of PA and H19 on cellular Pink1 mRNA levels detected by RT-qPCR. No statistically significant differences were found among the four groups. **B** Results of RAP using H19 probes. H19 probes were divided into two groups: odd and even, and Lac Z was used as an external reference. Lane 1 represents the marker (500, 400, 300, 200, 150, 100); lanes 2, 6, and 10: inputs for H19, Pink1, and GAPDH, respectively; lanes 3, 7, and 11: even groups of H19, Pink1, and GAPDH, respectively; lanes 4, 8, and 12: odd groups of H19, Pink1, and GAPDH, respectively; lanes 5, 9, and 13: negative control groups of H19, Pink1, and GAPDH, respectively. RT-qPCR was carried out to verify that H19 and Pink1 mRNA could be detected in the molecular complex precipitated using H19 probes. **C** RT-qPCR detecting RNA of H19 and Pink1 in the products yielded by RAP using different H19 probes. **D** Result of LC-MS/MS using proteins yielded by RAP using H19 probe. Bar charts showed the classification of proteins and numbers of included genes. **E** Western blot detecting the presence of eIF4A2 in the proetins yielded by RAP using H19 probe. **F,** **G** RT-qPCR detecting the presence of Pink1 and H19 in the RNA yielded by RIP using eIF4A2 antibody. Statistical analyses were performed base on three biological replicates. Data in **B** are expressed as mean ± SEM. *P < 0.05; **P < 0.01; ***P < 0.001. RT-qPCR, quantitative reverse transcription polymerase chain reaction; NC, negative control; RAP-MS, RNA antisense purification- mass spectrum; eIF4A2, eukaryotic translation initiation factor 4A, isoform 2; RIP, RNA-binding protein immunoprecipitation.

The aforementioned finding that H19 did not alter intracellular Pink1 mRNA levels did not support the involvement of the transcription mechanism in this regulation process (Fig. 5A). Therefore, we speculated the direct binding of H19 with Pink1. To test this hypothesis, we performed RNA antisense purification (RAP) and proteins were subjected to liquid chromatography with tandem mass spectrometry (LC-MS/MS). However, we could not find Pink1 protein in the results of LC-MS/MS. We next explore the binding between H19 and Pink1 mRNA and results were shown in Fig. 5B, C. As shown, Pink1 mRNA were significantly enriched in the RNA precipitated using an H19 probe. To explore the potential proteins involved in the process of H19-mediated regulation of Pink1, we continued to search proteins analyzed by LC-MS/MS. The competing endogenous RNA is the most widely investigated of the interactions between lncRNA and mRNA, but the key protein, ago2, was not found in the proteins precipitated by H19. However, the nucleic acid-binding proteins were mostly enriched (Fig. 5D). Among all the nucleic acid-binding proteins, we found that the key protein for mRNA translation, eIF4A2, was highly enriched by the H19 probe; this result was further validated using western blotting (Fig. 5E). RNA-binding protein immunoprecipitation (RIP) results using anti-eIF4A2 antibody demonstrated that both H19 and Pink1 mRNA bound to eIF4A2 (Fig. 5F, G).

### PA promotes Dnmt3b activity and suppresses H19 expression

**Fig. 6:**
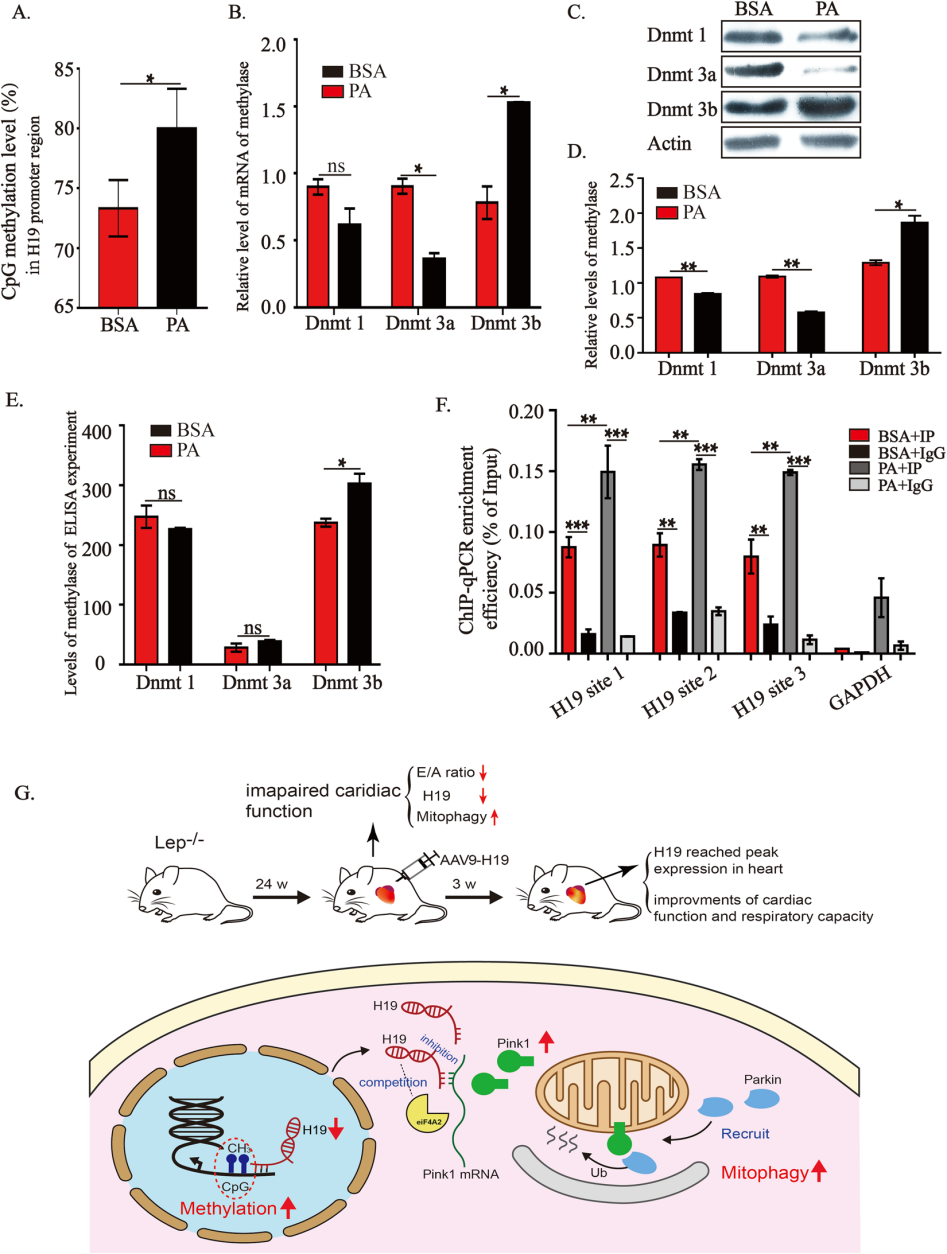
PA promotes Dnmt3b activity and suppresses H19 expression. **A** Effects of PA on CpG island methylation levels in H19 promoter region. **B**–**E** Effects of PA on the expressions of Dnmt1, Dnmt3a and Dnmt3b mRNA (**B**) and proteins (**C** and **D**), and DNA methylation activity (**E**). **F** ChIP analysis using the anti-Dnmt3b antibody. **G** Schematic diagram representing the potential mechanism underlying H19-mediated regulation of mitophagy during obesity. Data in **A**, **B** and **D**–**F** are expressed as mean ± SEM. *P < 0.05; **P < 0.01; ***P < 0.001. BSA, bovine serum albumin; PA, palmitic acid; Dnmt1, Dnmt3a or Dnmt3b, DNA methyltransferase 1, 3a or 3b; ChIP, Chromatin immunoprecipitation; RT-qPCR, quantitative reverse transcription polymerase chain reaction.

The above data demonstrated the effects and underlying mechanisms of H19 on PA-induced inhibition of cardiac mitochondrial respiration. However, the mechanism by which PA regulates H19 remains to be investigated. Since PA is widely accepted to regulate gene expression in an epigenetic manner and the expression of H19, as an imprinted gene, is markedly influenced by epigenetic modification^26–28^, we explored whether there were any PA-mediated changes in DNA methylation within the H19 promoter region. As shown in Fig. 6A, enhanced DNA methylation in the H19 promoter sequence was observed, as manifested in terms of CpG methylation, in PA-treated cells. Three main DNA methyltransferases (Dnmt), namely Dnmt1, Dnmt3a, and Dnmt3b^29^, determine differentially methylated regions (DMRs), which are crucial in the regulation of imprinted gene expression. Thus, we investigated the expression and DNA methyltransferase activity of these proteins. There was a significant increase in the mRNA levels of Dnmt3b upon PA treatment, while those of Dnmt3a was reduced and Dnmt1 remained unaltered (Fig. 6B). The protein levels of Dnmt3b were also elevated upon PA treatment, while those of Dnmt3a and Dnmt1 remained unchanged (Fig. 6C, D). Determination of Dnmt activity using ELISA showed increased Dnmt3b activity, with no change in the activities of Dnmt3a and Dnmt1 (Fig. 6E). In continuation of these results, chromatin immunoprecipitation **(**ChIP) was performed using an anti-Dnmt3b antibody, the results for which are shown in Fig. 6F. As shown, DNA fragments containing putative CpG sites in the H19 promoter region were all significantly precipitated by the anti-Dnmt3b antibody.

Taken together, these results clearly demonstrated that PA increased Dnmt3b expression and activity, which induces CpG site methylation in the H19 promoter region, eventually leading to the inhibition of H19 transcription.

## Discussion

The deleterious effects of obesity on cardiac structure and function are well recognized, but the detailed mechanisms underlying these effects still yet to be clarified. In the present study, we concluded that H19-mediated Pink1 regulation is critically involved in obesity-induced mitochondrial suppression and cardiac dysfunction. Moreover, we proposed that mitophagy, which is usually believed to be a protective and adaptive mechanism in pathological conditions, is hyperactive in this situation.

The current study supports the enhancement of mitochondrial respiration as a solution for relieving obesity-induced cardiac dysfunction. Under normal conditions, fatty acids account for ~70% of the substrates for energy production in the heart. In the setting of obesity, due to in increased fatty acids and decreased capacity to utilize glucose, fatty acids may serve as up to 90% of the energy substrates for cardiomyocyte metabolism. Mitochondria are the main sites for the metabolism of these increased levels of intracellular fatty acids, and oxidation is the major mechanism for such utilization. There is a consensus that increased fatty acid oxidation in obesity compromises cardiac function^30,31^, but the solutions to reduce the excessive fatty acid levels are highly debated. Studies have suggested that the adoption of various fatty acid oxidation antagonists are beneficial^30^. In contrast, recent studies using a genetic model that promotes fatty acid oxidation yielded robust protective effects in high-fat diet-fed mice^16^. The most plausible explanation for this discrepancy could be the amount of residual mitochondrial mass inside the cells. In advanced cardiac impairment, mitochondrial capacity is markedly reduced, and the inhibition of fatty oxidation rate could alleviate the oxidative burden of the mitochondria, thus prolonging cell survival. However, in the settings with mild to moderate mitochondrial damage, such as obesity and early stage of diabetes without other established primary cardiac insults, accelerating fatty acid may help reduce the levels of intracellular redundant lipids and relieve peroxidation.

Similar contrasts have been observed in the manipulation of mitophagy. The present study demonstrated that counteracting mitophagy using siRNA against Pink1 resulted in improved mitochondrial respiration, which implied the occurrence of excessive mitophagy. This coincides with the finding that excessive mitophagy leads to insufficient energy production and apoptosis in vascular smooth muscle cells within advanced atherosclerotic plaques^18^. However, mitophagy cannot be completely abolished, and moderate mitophagy is important for cardiac structure and function^16,32^. In the present study, we also found that reduced mitochondrial membrane potential upon PA treatment implies a need for mitophagy to some extent for clearing damaged mitochondria. Notably, early and mild obesity or saturated fatty acid burden enhances cardiac mitophagy^33^, while the late stage of obesity and heavy fatty acid stimulus inhibits mitophagy^16^.

With regard to the pathways triggering mitophagy, the Parkin pathway has been reported to be downregulated in hearts of both genetic obesity and high-fat-fed mice^16,32^. In contrast, other studies have shown an increasing trend of Parkin-dependent mitophagy in high-fat diet-fed mice^33,34^. With respect to the Parkin-independent mechanisms, Fundc1-induced mitophagy has been reported to be involved in alleviating dietary-induced obesity and metabolic syndrome^35^, but the role of Bnip3-related mitophagy seems to be unfavorable for high-fat diet-fed mice^36^. These results also indicate the complexity of the involvement of mitophagy in fatty acid loading, thus highlighting the need for further studies.

H19 expression is highly regulated by DMR methylation and the nucleic acid sequences of H19 are highly conserved among species^37^. In the cardiac settings, H19 confers protection against left ventricular hypertrophy and ischemia^37,38^. However, H19 has been shown to play an unfavorable role in right ventricular hypertrophy^39^. In hepatic, adipose, and β-cells, H19 counteracts obesity and relieves insulin resistance^40–42^. Although these effects of H19 have been reported, to the best of our knowledge, the current study is the first to demonstrate the effects of H19 expression in vivo, especially in cardiomyocytes or H9c2 cells, on obesity-associated cardiac damage.

Results from studies using different cell types support that H19 is mainly, even exclusively, expressed in the cytoplasm^43–45^. Based on this subcellular location, competing endogenous RNA regulation is the most reported mechanism of action of H19, followed by direct binding to transcription regulators or other proteins^46,47^. Currently, few study have investigated the role of H19 in regulating mRNA translation. H19 has been predicted to bind to eIF4A3^48^. However, this is not supported by the LC-MS/MS results in the current study, in which did eIF4A3 colud not be found. Instead, eIF4A2 was found to be significantly enriched in proteins obtained by RAP with H19 probes. This seems reasonable because eIF4A3 is mainly located inside the nucleus, whereas eIF4A2 is located largely in the cytoplasm. Based on our demonstration that Pink1 protein, rather than mRNA, was negatively regulated by H19 and that the complex of H19, Pink1 mRNA, and the crucial mRNA translation regulator, eIF4A2, were robustly validated using RAP and RIP, it may be plausible to infer that H19 regulates Pink1 expression by inhibiting mRNA translation.

The current study has some limitations. First, despite the potential effects and underlying mechanisms of H19 in counteracting obesity-induced mitochondrial and cardiac dysfunctions in vivo and in vitro, Parkin-related mitophagy in this situation, as stated previously, has already been reported and this part may not be attractive enough. Second, we did not detect these effects of H19 in the late stage of obesity, wherein mitophagy is reduced rather than enhanced. Thus, the protective effects of H19 cannot be directly extended to these situations. Third, we did not perform Pink1 rescue experiments following H19-induced mitophagy suppression. Although Pink1/Parkin is the most classical signaling pathway for mitophagy, due to the lack of experimental results for such rescue experiments, the involvement of other mitophagy pathways in H19-induced mitophagy regulation cannot be entirely excluded.

In summary, despite the limitations, the current study demonstrated that reduced H19 levels were involved in obesity-induced cardiac dysfunction, and the underlying mechanisms were also elucidated. Comprehensive studies that investigate more mitophagy-related pathways as well as the different stages of cardiac insults are highly warranted to better elucidate the relationships among H19, mitophagy, mitochondrial respiration, and cardiac function.

## Materials and methods

### Animal experiments

All animal procedures were performed in conformance with the Guide for the Care and Use of Laboratory Animals published by the US National Institutes of Health and approved by the Institutional Animal Care and Use Committee of Sun Yat-sen University (SYSU-IACUC-2020-000235). Seven-week-old Lep^−/−^ and WT mice were purchased from the Model Animal Research Center of Nanjing University. All mice were housed at 25 °C and fed a regular diet purchased from Guangdong Province Medical Animal Centre under standard conditions (12-h/12-h light/dark cycle).

The mice were anesthetized with 1% isoflurane and subjected to echocardiography, following which echocardiograms were obtained and analyzed using the Vevo^®^ 2100 Imaging System (VisualSonics Inc.) and MicroScan™ Transducer (MS-400, 30 MHz, VisualSonics Inc.). LVDd, E/A ratios, and ejection fraction values were measured as previously reported^9^.

### Cell culture, transfection, and adeno-associated virus/lentivirus construction

Embryonic rat heart H9c2 cells were obtained from ATCC^®^ (CRL-1446™, Manassas, USA). Dulbecco’s Modified Eagle Medium (DMEM) (catalog number C11995500BT, Gibco, California, USA) supplemented with 10% Fetal Bovine Serum (FBS) (catalog number 16140071, Gibco, California, USA) and 1% penicillin/streptomycin (catalog number 15140122, Gibco, California, USA) was used to culture H9c2 cells at 37 °C in a humidified atmosphere incubator containing 5% CO_2_. PA (catalog number P0500, Sigma-Aldrich, Missouri, USA) and fatty acid-free bovine serum albumin (BSA) (catalog number A8850, Solarbio, Beijing, China) conjugates were prepared by soaking PA with sodium hydroxide and mixing it with fatty acid-free BSA. PA conjugates were diluted in 10% FBS-containing culture medium and incubated with H9c2 cells for about 24 h^9^. BafA1 (catalog number S1413, Selleck, Texas, USA) at a concentration of 50 nM was used as a mito-lysosomal inhibitor to verify the main mechanism for mitochondrial losing.

siRNA against Pink1 mRNA, provided by IGE Biotechnology (Guangzhou, China), was used to evaluate the role of Pink1-initiated mitophagy under PA-treated conditions. siRNA transfection was performed using Lipofectamine^®^ RNAiMAX reagent (catalog number 13778150, Life Technologies, California, USA). Fifty microliters of Opti-MEM (catalog number 11058021, Life Technologies, California, USA) was used to dilute one microliter of siRNA and three microliters of Lipofectamine^®^ RNAiMAX, according to the manufacturer’s instructions. The transfection complexes were incubated in complete medium for ~18 h. The mixed medium was then replaced with regular complete medium for another 48 h of incubation. The detailed sequences of the siRNAs are listed in Supplemental material. 1. Validation of RNA interference efficiency is shown in Sup. Fig. 2J.

AAV 2/9 was used to express H19 under the control of the cTnT promoter for in vivo study. The sequence of the cTnT promoter was obtained from Addgene (Massachusetts, USA), which was used to replace the original promoter of the AAV expression vector pAAV-CMV-H19. DNA sequences for mouse H19 transcription (GenBank number 14955) were synthesized by OBiO Biology (Shanghai, China), which were then cloned downstream of the cTnT promoter. Green fluorescent protein was used as a control. AAV package transfections were performed as previously described. Briefly, expression vectors and package vectors were transfected into 293AAV cells using a calcium phosphate-mediated protocol, as previously described. Cells were harvested 72 h later, and AAVs were purified from them using cesium chloride (CsCl) gradient centrifugation. AAVs were used at a concentration of 2.5 × 10^11^ vg/mL per mouse.

Lentiviruses were used to express mitoTimer and mtKeima in cells to track mitochondrial turnover and mitophagy, as described in previous studies^49,50^. To avoid increased mitochondrial burden due to consistent expression, we adopted an inducible expression approach—the Tet-On expression system^49^—to express mitoTimer and mtKeima. Briefly, the coding sequences of mtKeima and mitoTimer were obtained from Addgene (mitoTimer code: 50547^49^, mtKeima code: 72342^50^), which were cloned into the expression vector of the Tet-On system (pLVX-TetOne-Puro, Clontech, Japan). All the clones were sequenced and verified. For lentivirus packages, the expression vectors and packaging vectors were transfected into 293T cells using the same approach as in case of the AAV package. After 72 h of incubation, the supernatant was collected, and the lentivirus was purified using CsCl gradient centrifugation. Lentiviruses were used at a multiplicity of infection (MOI) of 75 for all the experiments. Puromycin (1 μg/mL) was used to screen stably expressing cells, and doxycycline (2 μg/mL) was used to induce target protein expression.

### Flow cytometry analysis

Staining with MitoTracker™ Green FM (catalog number M7514; Thermo Fisher Scientific, California, USA) was used to quantify the number of mitochondria. The stock solution was diluted to a working concentration of 50 nM with culture medium and incubated for 45 min at 37 °C. The samples were then washed and subjected to flow cytometry at an excitation wavelength of 490 nm.

JC-1 probes assay kit (catalog number C2006; Beyotime, Shanghai, China) was used to evaluate mitochondrial membrane potential. The JC-1 stock solution was diluted to working solution with buffer solution according to manufacturer’s manual. In all, 2 × 10^6^ cells of each sample were harvested and incubated with JC-1 working solution for 20 min at 37 °C. Washing samples for three times and conducted flow-cytometric analysis.

### Fluorescence detection

Immunofluorescence (for LC3B-II, Tom20, and LAMP1 detection) and fluorescence intensity of mtKeima and mitoTimer was observed under a fluorescence microscope to assess mitophagy. For immunofluorescence staining, samples were pre-treated as described above, fixed in 4% paraformaldehyde, and permeabilized with PBS-Triton (0.4%). Subsequently, the samples were incubated overnight with primary antibodies against LC3B‐II, Tom20, and LAMP1. Next, the samples were rinsed and incubated with a secondary antibody in a humidified container for 1 h at 37 °C. Finally, the samples were stained with DAPI. Random fields of view were selected, and each group was arranged for several biological duplications. The primary and secondary antibodies used in this study are listed in Supplemental material. 2.

mtKeima was detected at an excitation wavelength of 458 nm at neutral pH (autophagosome) and an excitation wavelength of 561 nm at an acidic pH (autolysosome) using confocal microscopy- Zeiss LSM 800^50^. The red intensity area and total intensity area (red and green intensity area) of mtKeima proteins were evaluated using Zeiss ZEN 2.3 imaging software. MitoTimer shifts its color as the protein matures, and thus, its fluorescence shifts over time from green (excitation wavelength = 483 nm) to red (excitation wavelength = 558 nm)^51^.

### Transmission electron microscopy detection

TEM was used to observe autophagosomes and mitochondria in vivo and in vitro. Small cubic pieces of cardiac tissue (~1 mm^3^) or cell samples were pre-treated as described above and fixed with 2.5% glutaraldehyde and 1% osmium tetroxide. The embedded samples were then impregnated with the epoxy-dendritic ester, epon-812. The samples were subsequently sectioned and double-stained with uranium acetate and lead citrate. Finally, autophagosomes and mitochondria were observed using TEM, at random fields of view.

### Seahorse experiments

Seahorse was used to detect mitochondrial respiratory capacity as described in our previous study^52^. The Seahorse XF Cell Mito Stress Test Kit (catalog number 103015-100, Agilent, Delaware, USA) was used to directly measure the oxygen consumption rate (OCR) using an Agilent Seahorse Xfe96 Extracellular Flux Analyzer (Agilent, Delaware, USA)^53^. First, cells (1 × 10^4^ cells/well) were seeded onto Seahorse XF cell culture 96-well microporous plates (catalog number 103729-100, Agilent, Delaware, USA) and treated as described above. Seahorse XF calibration solution was then used to hydrate the sensor probe plate at 37 °C in a CO_2_-free incubator overnight. Next, three compounds, oligomycin A, FCCP, and antimycin A at final concentrations of 1.5, 1, and 0.5 μM, respectively, were added to each well, followed by detection of the OCR according to the manufacturer’s instructions.

### Western blotting and quantitative reverse transcription polymerase chain reaction

Western blotting was carried out to detect protein expression in the cardiac tissues, cells, or mitochondria. Mitochondria were isolated using a Mitochondria Isolation Kit (catalog number 89874, Thermo Fisher Scientific, California, USA), according to the manufacturer’s instructions. Briefly, 2 × 10^7^ cells were harvested from each sample, followed by the addition of 800 µL of Mitochondria Isolation Reagent A into each tube. The cells were then vortexed at medium speed for 5 s and incubated on ice for 2 min. Next, 800 µL of Mitochondria Isolation Reagent C was added to the samples, following which the mitochondrial pellets were harvested (validation of mitochondrial isolation efficiency is presented in Sup. Fig. 2F). Left ventricular tissues were homogenized with RIPA buffer (catalog number 9086S, Cell Signaling Technology, Massachusetts, USA; 1 mL RIPA buffer was used to lyse 100 mg cardiac tissue) to obtain the protein samples. The cellular and mitochondrial pellets were also lysed with RIPA buffer. Samples from the homogenized cardiac tissue, cellular lysate, and isolated mitochondrial pellets were prepared for western blotting; SDS-PAGE was performed using three biological replicates. Following SDS-PAGE, the protein samples were transferred to 0.2- or 0.45-micrometer PVDF membranes (catalog number ISEQ00010/IPVH00010, Millipore, Massachusetts, USA), and then blocked and probed with primary antibodies overnight at 4 °C. Next, the membranes were incubated with an anti-rabbit/mouse secondary antibody for another hour. The protein bands were detected using chemiluminescence with an ECL system (catalog number WBULS0500, Merck Millipore, Darmstadt, Germany). The intensities of the bands were quantified using ImageJ software (National Institutes of Health) and normalized to those of the control. The primary and secondary antibodies used in this study are listed in Supplemental material. 2.

Left ventricular tissues were homogenized and treated with RNAiso plus (catalog number 9109, Takara, Shiga, Japan; 1 mL Trizol™ was used for 100 mg cardiac tissue) and subjected to a round of centrifugation to obtain samples for RNA extraction. RNA from cardiac and cellular samples was obtained using the RNeasy Mini Kit (catalog number 74106, Qiagen, California, USA), and RT-qPCR was conducted. Briefly, PrimeScript™ RT Master Mix (catalog number RR036A, Takara, Shiga-ken, Japan) was used to perform RNA reverse transcription using a Light Cycler^®^ 96 Real-Time PCR System (Roche, Basel, Switzerland). The expression levels were normalized to those of Tubulin, and the data were analyzed using the 2^−ΔΔCt^ method. All the primers used in this study are listed in Supplementals material. 3.

mtDNA copy numbers were calculated to quantify the number of mitochondria. This experiment was conducted with FastStart™ Essential DNA Green Master (catalog number 06402712001, Roche, Basel, Switzerland) on a Roche LightCycler^®^ 480 System. For each group, 1 × 10^7^ cells were harvested and lysed in 200 μL lysis buffer (catalog number R0020, Solarbio, Beijing, China). The cell lysate was subjected to a round of centrifugation, following which the supernatant was transferred to new tubes. The following cycling parameters were used: 95 °C for 10 min, followed by 45 cycles of 95 °C for 15 s and 60 °C for 45 s. All the primers used in this study are listed in Supplemental material. 3.

### RNA antisense purification, RNA-binding protein immunoprecipitation, and chromatin immunoprecipitation assays

The RAP (catalog number Bes1009), RIP (catalog number Bes1007), and ChIP kits (catalog number Bes5001) were purchased from BersinBio (Guangzhou, China). The RAP assay was performed to investigate the potential binding of proteins or RNA to H19. The general steps were as follows: H9c2 cells (~6 × 10^7^ cells) were harvested and washed several times. DNase was added to the cells, following which the cells were incubated with streptavidin magnetic bead-conjugated RNA probes. The H19 probes in our experiment were divided into two groups according to their positions corresponding to the H19 sequence. Lac Z probes were used as the control. Next, the compounds pulled down by the probes were subjected to RT-qPCR and western blotting, as described above.

The RIP assay was used to verify the binding of RNA to eIF4A2 using an anti-eIF4A2 antibody. First, nuclear RNA was extracted, and protein A/G beads were prepared for each sample. Next, anti-eIF4A2 antibody and an equal quantity of IgG were added to the nuclear RNA in the IP and IgG groups, respectively, and incubated in a vertical mixer overnight at 4 °C. Next, protein A/G beads were added to the IP and IgG groups, and the samples were mixed in a vertical mixer for 2 h at 4 °C, following which the beads were collected and the supernatant was discarded. Subsequently, polysome elution buffer was added to the IP and IgG groups, and RNA was obtained from the groups using TRIzol™ reagent. The levels of H19 and Pink1 mRNA were verified using RT-qPCR, as described above.

ChIP assay was conducted to verify the binding of Dnmt3b to the H19 promoter region. Briefly, 2 × 10^7^ cells were harvested and cross-linked with 1% formaldehyde. The samples were then neutralized for ~5 min, followed by washing and pellet collection. ChIP was carried out using an anti-Dnmt3b antibody, according to the manufacturer’s instructions. Anti-Dnmt3b antibody and isotope IgG were applied to the immunoprecipitated DNA, and RT-qPCR was conducted as described above to analyze the binding of Dnmt3b to the H19 promoter region.

### Statistical analysis

The data obtained are represented as mean ± SEM. One-way analysis of variance was used to compare the different groups. A paired *t*-test was used to compare the echocardiograph parameters between the different treatment groups of mice. All data were analyzed using Prism 7 (GraphPad, San Diego, CA, USA). Differences were considered statistically significant at *p* < 0.05.

## Supplementary information

Supplemental materials

## References

[1] Twig G (2016). Body-mass index in 2.3 million adolescents and cardiovascular death in adulthood. N. Engl. J. Med.

[2] Ma C (2017). Effects of weight loss interventions for adults who are obese on mortality, cardiovascular disease, and cancer: systematic review and meta-analysis. BMJ.

[3] Robertson J (2020). Body mass index in young women and risk of cardiomyopathy: a long-term follow-up study in Sweden. Circulation.

[4] Mouton AJ, Li X, Hall ME, Hall JE (2020). Obesity, hypertension, and cardiac dysfunction: novel roles of immunometabolism in macrophage activation and inflammation. Circ. Res..

[5] Lavie CJ, Pandey A, Laum DH, Alpert MA, Sanders P (2017). Obesity and atrial fibrillation prevalence, pathogenesis, and prognosis: effects of weight loss and exercise. J. Am. Coll. Cardiol..

[6] Wang N (2020). HSF1 functions as a key defender against palmitic acid-induced ferroptosis in cardiomyocytes. J. Mol. Cell Cardiol..

[7] Wang LY (2020). Soluble epoxide hydrolase deficiency attenuates lipotoxic cardiomyopathy via upregulation of AMPK-mTORC mediated autophagy. J. Mol. Cell Cardiol..

[8] Sletten AC, Peterson LR, Schaffer JE (2018). Manifestations and mechanisms of myocardial lipotoxicity in obesity. J. Intern. Med..

[9] Wu MX (2020). Interleukin-33 alleviates diabetic cardiomyopathy through regulation of endoplasmic reticulum stress and autophagy via insulin-like growth factor-binding protein 3. J. Cell Physiol..

[10] Zhong P (2017). Role of CaMKII in free fatty acid/hyperlipidemia-induced cardiac remodeling both in vitro and in vivo. J. Mol. Cell Cardiol..

[11] Ashrafi G, Schwarz TL (2013). The pathways of mitophagy for quality control and clearance of mitochondria. Cell Death Differ..

[12] Sciarretta S, Maejima Y, Zablocki D, Sadoshima J (2018). The role of autophagy in the heart. Annu. Rev. Physiol..

[13] Wallace KB, Sardão VA, Oliveira PJ (2020). Mitochondrial determinants of doxorubicin-induced cardiomyopathy. Circ. Res.

[14] Gustafsson ÅB, Dorn GW (2019). Evolving and expanding the roles of mitophagy as a homeostatic and pathogenic process. Physiol. Rev..

[15] Law BA (2018). Lipotoxic very-long-chain ceramides cause mitochondrial dysfunction, oxidative stress, and cell death in cardiomyocytes. FASEB J..

[16] Shao D (2020). Increasing fatty acid oxidation prevents high-fat diet-induced cardiomyopathy through regulating parkin-mediated mitophagy. Circulation.

[17] Kubli DA, Gustafsson ÅB (2012). Mitochondria and mitophagy: the yin and yang of cell death control. Circ. Res..

[18] Yu EPK (2017). Mitochondrial respiration is reduced in atherosclerosis, promoting necrotic core formation and reducing relative fibrous cap thickness. Arterioscler. Thromb. Vasc. Biol..

[19] Li X (2019). lncRNA H19 alleviated myocardial i/ri via suppressing miR-877-3p/Bcl-2-mediated mitochondrial apoptosis. Mol. Ther. Nucleic Acids.

[20] Liu Y (2020). Ghrelin protects against obesity-induced myocardial injury by regulating the lncRNA H19/miR-29a/IGF-1 signalling axis. Exp. Mol. Pathol..

[21] Ye WC, Huang SF, Hou LJ, Zhao GJ (2020). Blocking lncRNA H19/miR-194-5p/SIRT1 axis in cardiac myocyte is responsible for doxycycline inhibiting autophagy. Int. J. Cardiol..

[22] Lazarou M (2015). The ubiquitin kinase PINK1 recruits autophagy receptors to induce mitophagy. Nature.

[23] Shin WH, Chung KC (2020). Human telomerase reverse transcriptase positively regulates mitophagy by inhibiting the processing and cytoplasmic release of mitochondrial PINK1. Cell Death Dis..

[24] Jin GX (2018). Atad3a suppresses Pink1-dependent mitophagy to maintain homeostasis of hematopoietic progenitor cells. Nat. Immunol..

[25] Yu LM (2021). Melatonin attenuates diabetic cardiomyopathy and reduces myocardial vulnerability to ischemia-reperfusion injury by improving mitochondrial quality control: Role of SIRT6. J. Pineal Res..

[26] Martinet C (2016). H19 controls reactivation of the imprinted gene network during muscle regeneration. Development.

[27] Nordin M, Bergman D, Halje M, Engström W, Ward A (2014). Epigenetic regulation of the Igf2/H19 gene cluster. Cell Prolif..

[28] Hadji F (2016). Altered DNA methylation of long noncoding RNA H19 in calcific aortic valve disease promotes mineralization by silencing NOTCH1. Circulation.

[29] Brant JO, Riva A, Resnick JL, Yang TP (2014). Influence of the Prader-Willi syndrome imprinting center on the DNA methylation landscape in the mouse brain. Epigenetics.

[30] Lopaschuk GD, Ussher JR, Folmes CD, Jaswal JS, Stanley WC (2010). Myocardial fatty acid metabolism in health and disease. Physiol. Rev..

[31] Fillmore N, Mori J, Lopaschuk GD (2014). Mitochondrial fatty acid oxidation alterations in heart failure, ischaemic heart disease and diabetic cardiomyopathy. Br. J. Pharm..

[32] Ren J (2020). FUNDC1 interacts with FBXL2 to govern mitochondrial integrity and cardiac function through an IP3R3-dependent manner in obesity. Sci. Adv..

[33] Tong MM (2019). Mitophagy is essential for maintaining cardiac function during high fat diet-induced diabetic cardiomyopathy. Circ. Res..

[34] Dhanabalan K, Mzezewa S, Huisamen B, Lochner A (2020). Mitochondrial oxidative phosphorylation function and mitophagy in ischaemic/reperfused hearts from control and high-fat diet rats: effects of long-term melatonin treatment. Cardiovasc. Drugs Ther..

[35] Wu H (2019). Deficiency of mitophagy receptor FUNDC1 impairs mitochondrial quality and aggravates dietary-induced obesity and metabolic syndrome. Autophagy.

[36] da Silva Rosa, S. C. et al. BNIP3L/Nix-induced mitochondrial fission, mitophagy, and impaired myocyte glucose uptake are abrogated byPRKA/PKA phosphorylation. Autophagy (2020). 10.1080/15548627.2020.1821548. 10.1080/15548627.2020.1821548 PMC8496715 33044904

[37] Viereck J (2020). Targeting muscle-enriched long non-coding RNA H19 reverses pathological cardiac hypertrophy. Eur. Heart J..

[38] Huang PS (2020). Atorvastatin enhances the therapeutic efficacy of mesenchymal stem cells-derived exosomes in acute myocardial infarction via up-regulating long non-coding RNA H19. Cardiovasc. Res..

[39] Omura J (2020). Identification of long noncoding RNA H19 as a new biomarker and therapeutic target in right ventricular failure in pulmonary arterial hypertension. Circulation.

[40] Wang H (2020). Long non-coding RNA (lncRNA) H19 induces hepatic steatosis through activating MLXIPL and mTORC1 networks in hepatocytes. J. Cell Mol. Med..

[41] Schmidt E (2018). LincRNA H19 protects from dietary obesity by constraining expression of monoallelic genes in brown fat. Nat. Commun..

[42] Sanchez-Parra C (2018). Contribution of the long noncoding RNA H19 to β-cell mass expansion in neonatal and adult rodents. Diabetes.

[43] Zhou J (2019). Combined single-cell profiling of lncRNAs and functional screening reveals that h19 is pivotal for embryonic hematopoietic stem cell development. Cell Stem Cell.

[44] Runge S (2000). H19 RNA binds four molecules of insulin-like growth factor II mRNA-binding protein. J. Biol. Chem..

[45] Li JX (2020). Long non-coding RNA H19 regulates porcine satellite cell differentiation through miR-140-5p/SOX4 and DBN1. Front. Cell Dev. Biol..

[46] Raveh E, Matouk IJ, Gilon M, Hochberg A (2015). The H19 Long non-coding RNA in cancer initiation, progression and metastasis-a proposed unifying theory. Mol. Cancer.

[47] Zhang YH (2020). The lncRNA H19 alleviates muscular dystrophy by stabilizing dystrophin. Nat. Cell Biol..

[48] Han D (2016). Long noncoding RNA H19 indicates a poor prognosis of colorectal cancer and promotes tumor growth by recruiting and binding to eIF4A3. Oncotarget.

[49] Hernandez G (2013). MitoTimer: a novel tool for monitoring mitochondrial turnover. Autophagy.

[50] Sun N (2017). A fluorescence-based imaging method to measure in vitro and in vivo mitophagy using mt-Keima. Nat. Protoc..

[51] Ferree AW (2013). MitoTimer probe reveals the impact of autophagy, fusion, and motility on subcellular distribution of young and old mitochondrial protein and on relative mitochondrial protein age. Autophagy.

[52] Chen ZT (2020). Long non-coding RNA Linc00092 inhibits cardiac fibroblast activation by altering glycolysis in an ERK-dependent manner. Cell Signal.

[53] Divakaruni AS, Paradyse A, Ferrick DA, Murphy AN, Jastroch M (2014). Analysis and interpretation of microplate-based oxygen consumption and pH data. Methods Enzymol..

