# Supplementary material for: LncRNA H19 governs mitophagy and restores mitochondrial respiration in the heart through Pink1/Parkin signaling during obesity
Source: Cell Death Dis. 2021 May 28;12(6):557. doi: 10.1038/s41419-021-03821-6 (PMC8163878; doi:10.1038/s41419-021-03821-6)
Supplement: Supplementary file 1 — Supplemental materials [file 41419_2021_3821_MOESM1_ESM.docx]

**Table 1 siRNA sequences targeting Pink1**

| siRNA | Sense sequence | Antisense sequence |
| --- | --- | --- |
| Pink1-1 | CCUGGAGGAUUAUCUGAUA | UAUCAGAUAAUCCUCCAGG |
| Pink1-2 | GCCCAGAUGUCGUCUCAAA | UUUGAGACGACAUCUGGGC |
| Pink1-3 | GGCAGGAUCCUCCAGCGAA | UUCGCUGGAGGAUCCUGCC |

**Table 2 Antibodies used for Western blots analysis and immunofluorescence**

| Antibody name | Brand | Code | Dilution |
| --- | --- | --- | --- |
| anti-Pink1 | Novus | BC100-494 | 1:1000 (WB) |
| anti-Parkin | CST | 2123S | 1:1000 (WB) |
| anti-Phospho-Ubiquitin | CST | 62802S | 1:1000 (WB) |
| anti-Ubiquitin | Santa Cruz | sc-8017 | 1:500 (WB) |
| anti-Bnip3/Nix | CST | 12396 | 1:1000 (WB) |
| anti-Fundc1 | Abcam | ab224722 | 1:500 (WB) |
| anti-COXIV | CST | 4850 | 1:1000 (WB) |
| Total OXPHOS Rodent WB Antibody Cocktail | Abcam | ab110413 | 1:1000 (WB) |
| anti- PGC1 alpha | Abcam | ab54481 | 1:1000 (WB) |
| anti-TFAM | CST | 8076S | 1:1000 (WB) |
| anti-Tom20 | CST | 42406S | 1:1000 (WB) |
| anti-Tom20 | CST | 42406S | 1:200 (IF) |
| anti-Tom20 | Santa Cruz | sc-17764 | 1:100 (IF) |
| anti-Lamp1 | Santa Cruz | sc-20011 | 1:100 (IF) |
| anti-α-actin | CST | 6487 | 1:1000 (WB) |
| anti-α-Tubulin | CST | 2125S | 1:1000 (WB) |
| anti-LC3 | CST | 3868S | 1:1000 (WB) |
| anti-LC3 | CST | 3868S | 1:100 (IF) |
| anti-p62 | CST | 16177S | 1:1000 (WB) |
| anti-Dnmt3b | Bioss | bs-20680R | 1:1000 (WB) |
| anti-Dnmt3a | Bioss | bs-23029R | 1:1000 (WB) |
| anti-Dnmt1 | Bioss | bs-0678R | 1:1000 (WB) |
| anti-eiF4A3/2 | Abcam | ab180573/ab31218 | 1:1000 (WB) |
| anti-rabbit/mouse | CST | 7074S | 1:5000 (WB) |
| Goat Anti‐Mouse IgG H&L (Alexa Fluor® 488) | Abcam | ab150081 | 1:200 (IF) |
| Goat Anti‐Rabbit IgG H&L (Alexa Fluor® 488) | Abcam | ab150077 | 1:200 (IF) |
| Goat Anti‐Mouse IgG H&L (Alexa Fluor® 594) | Abcam | ab150116 | 1:200 (IF) |
| Goat Anti‐Rabbit IgG H&L (Alexa Fluor® 594) | Abcam | ab150080 | 1:200 (IF) |

Abbreviations: IF, immunofluorescence; WB, Western blots

**Table 3 Primer sequences used in RT-PCR**

| Primer name | Forward sequence | Reverse sequence |
| --- | --- | --- |
| Mouse H19 | ATCGGTGCCTCAGCGTTCGG | CTGTCCTCGCCGTCACACCG |
| Mouse GAPDH | AGGTCGGTGTGAACGGATTTG | TGTAGACCATGTAGTTGAGGTCA |
| Rat H19 | GGAAGGAGCATGGTGTGGTT | CTACACCTTCACTGCCCAGG |
| Rat Pink1 | ACTACCTATGCCCATCCATCTA | CTCGGTGACAGCTAAGTCATC |
| Rat Actin | TCAGGTCATCACTATCGGCAAT | AAAGAAAGGGTGTAAAACGCA |
| Rat Dnmt1 | AACCACCATCACATCTCATT | CATCTTCGTCCTCGTCAG |
| Rat Dnmt3a | GACCACGATAATACCTTCTCT | TGACGCTCTTCCTTACCA |
| Rat Dnmt3b | GCCGTTCTTCTGGATGTT | ACTTGGTGGTTATTGTCTGT |
| Rat Cytochrome B | GCCTCCGATTCATGTTAAGACTA | TACGCTATTCTACGCTCCATTC |
| Rat Beta-2-  microglobulin | CGACCGCACACTATAGGGAC | AGAGAACTCAACGGTGGCA |

Supplemental Figure 1


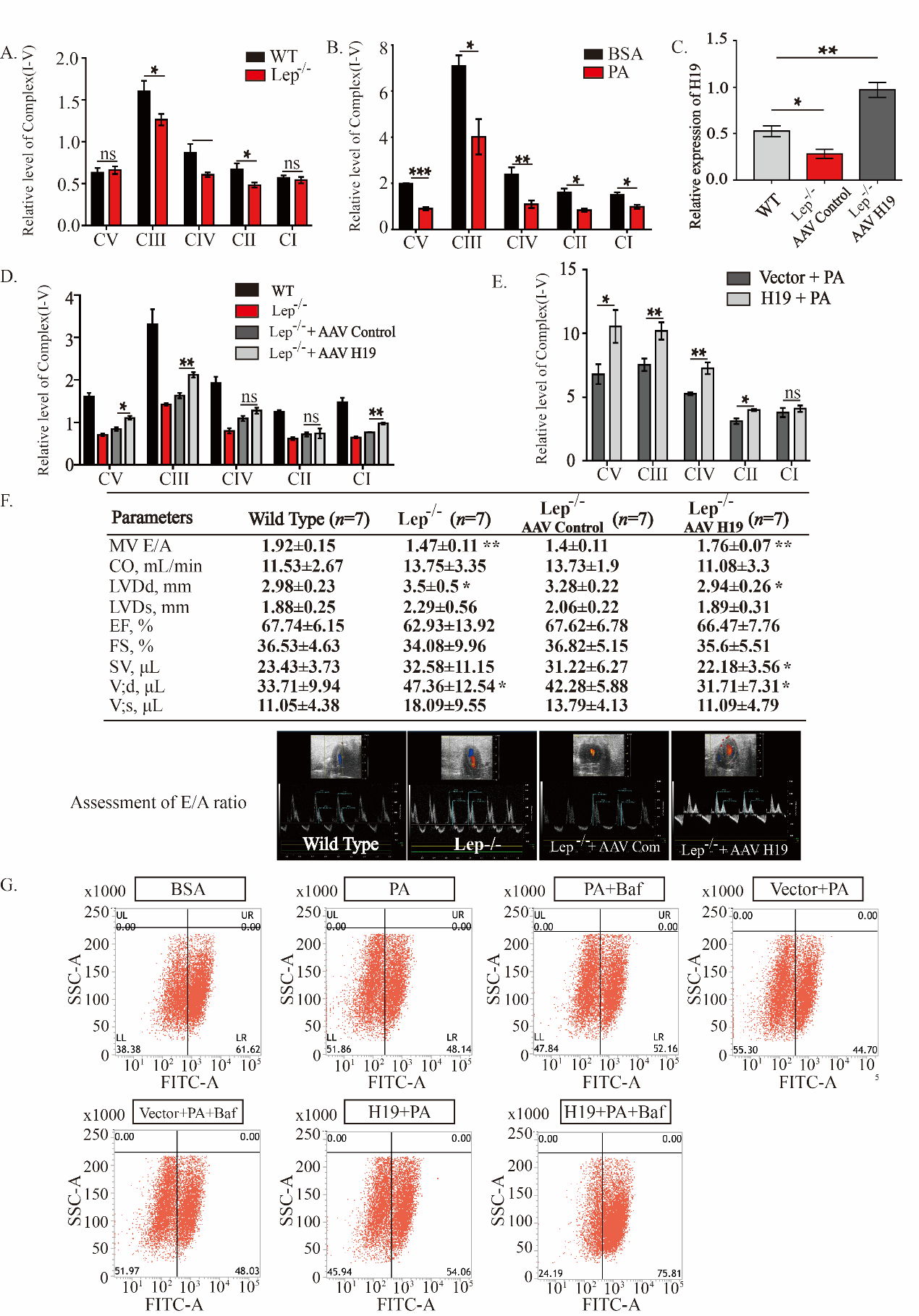


Supplemental Figure 2


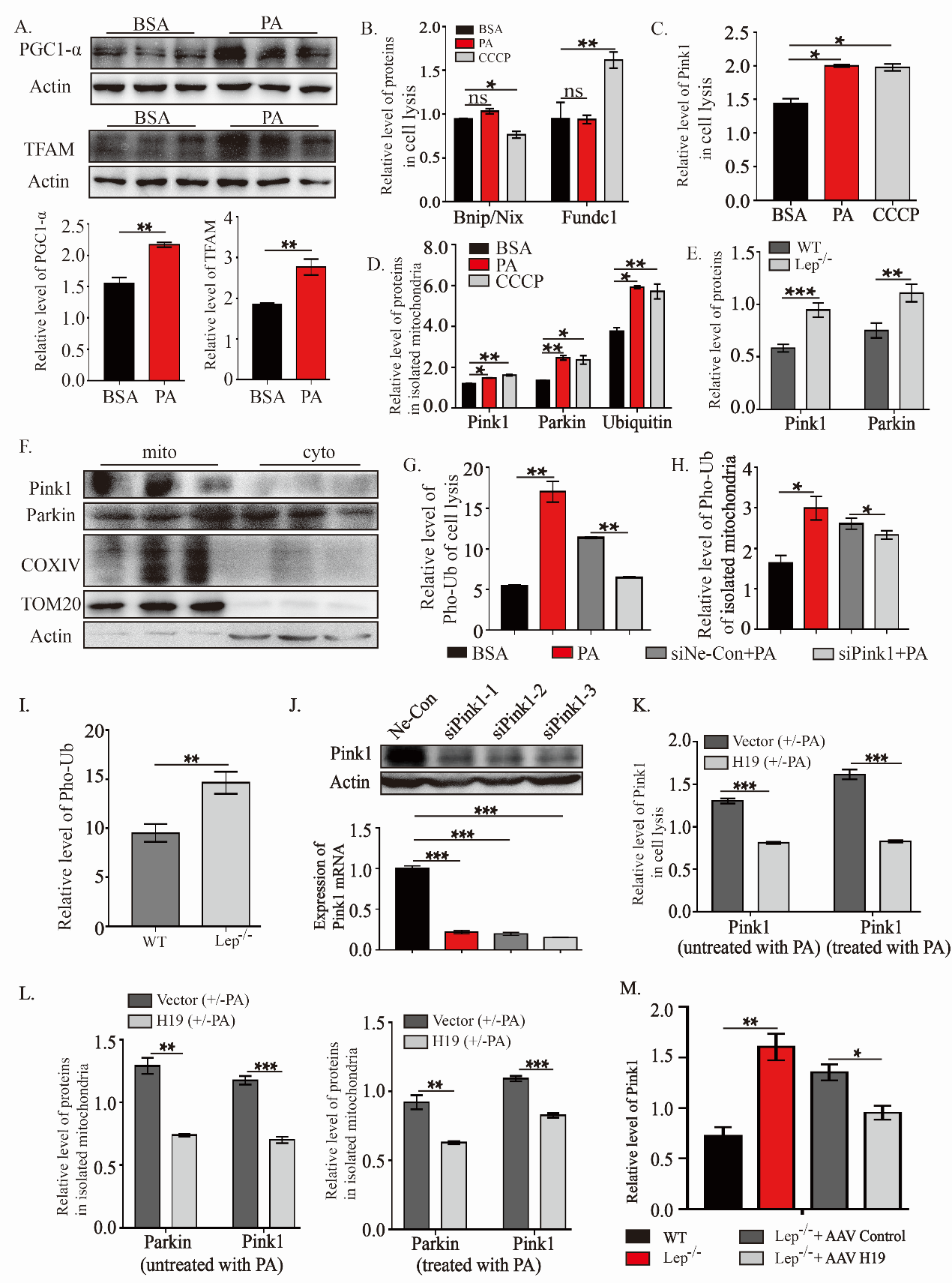


**Sup Fig. 1 A and B** Quantification of proteins for mitochondrial electron transport chain complexes (complexes I-V) in Fig. 1B and1C. **C** RT-qPCR for assessment of the efficiency of cardiac infectivity by AAV-H19. **D and E** Quantification of proteins for mitochondrial electron transport chain complexes (complexes I-V) in Fig. 1E and1F **F.** Results for echocardiography parameters of the four groups and corresponding echocardiography images of E and A waves. **G** Flow cytometry images of MitoTracker™ Green staining in Fig. 3N; FITC-A geometric mean was used to assess the mitochondrial fluorescence intensity. Data in **A-F** are expressed as mean±SEM. *, *P*<0.05; **, *P*<0.01; ***, *P*<0.001. BSA, bovine serum albumin; PA, palmitic acid; Lep^-/-^, B6-OB mice with leptin defects; WT, littermate control of Lep^-/-^; AAV, adeno-associated virus; CI-CV, mitochondrial respiratory chain complex I-V; H19, H19 overexpression; Vector, control lentivirus of H19; BafA1, bafilomycin A1.

**Sup Fig. 2 A** Western blotting for the detection of mitochondrial generation-related proteins PGC1-α and TFAM. **B-E** Gray scale analysis of western blot images in Fig. 4A-4D. **F** Validation of mitochondrial isolation efficiency. **G-I** Gray scale analysis of western blot images in Fig. 4F-4H. **J** Detection of Pink1 siRNA interference efficiency in Fig. 4F, 4G and 4L. **K-M** Gray scale analysis of western blot images in Fig. 4I-4K.*.* Data in **A-E** and **G-M** are expressed as mean±SEM. *, *P*<0.05; **, *P*<0.01; ***, *P*<0.001. BSA, Bovine Serum Albumin; PA, palmitic acid; CCCP，mitophagy inducer; PGC1-α, peroxisome proliferator-activated receptorγcoactivator-1α; TFAM, mitochondrial transcription factor A; WT, littermate control of Lep-/-; AAV, adeno-associated virus; si-Pink1, small interference RNA against Pink1; si-NE-Con，negative control of si-Pink1; AAV, adeno-associated virus; H19, H19 overexpression; Vector, control lentivirus of H19.
